# 

**Published:** 1888-08

**Authors:** 


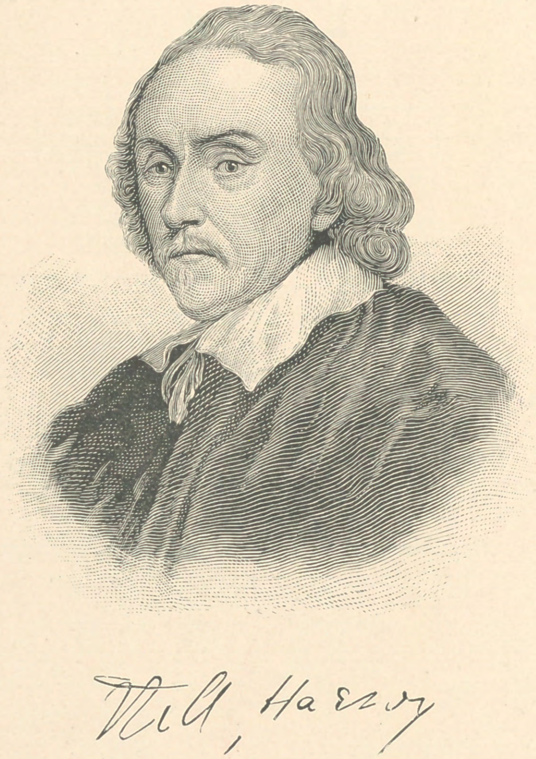

W. HARVEY, M. D.

DISCOVERER OF THE CIRCULATION OF THE BLOOD.

"The circling streams, once thought but pools of blood,
(Whether life's fuel, or the body's food)
From dark oblivion Harvey's name shall save."

Volume LVII.] AUGUST, 1888. [Number 2.

### The CHICAGO MEDICAL JOURNAL AND EXAMINER

EDITOR:
S. J. JONES, M.D., LL.D

COLLABORATORS:

CHICAGO:

PROFESSOR E. ANDREWS, M.D., LL.D.
PROFESSOR J. BARTLETT, M.D.
PROFESSOR W. T. BELFIELD, M.D.
PROFESSOR F. BILLINGS, M.D.
PROFESSOR W. H. BYFORD, A.M., M.D.
PROFESSOR L. CURTIS, A.M., M.D.
PROFESSOR N. S. DAVIS, JR., A.M., M.D.
PROFESSOR O. C. DEWOLF, A.M., M.D.
PROFESSOR E. C. DUDLEY, A.B., M.D.
PROFESSOR C. FENGER, M.D.
PROFESSOR J. N. HYDE, A.M., M.D.
PROFESSOR E. F. INGALS, A.M., M.D.
PROFESSOR F. S. JOHNSON, A.M., M.D.
PROFESSOR H. A. JOHNSON, M.D., LL.D.
PROFESSOR C. W. PURDY, M.D.
PROFESSOR C. G. SMITH, A.M., M.D.
PROFESSOR E. WING, A.M., M.D.

MILWAUKEE, WIS.:

PROFESSOR N. SENN, M.D., Ph.D.

WASHINGTON, D. C.:
S. O. RICHEY, M.D

UNITED STATES ARMY.
SURGEON D. L. HUNTINGTON, M.D.

UNITED STATES NAVY:
SURGEON-GENERAL J. M. BROWNE, M.D.
MEDICAL-DIRECTOR A. L. GIHON, A.M., M.D.

MARINE HOSPITAL SERVICE:
SURGEON S. T. ARMSTRONG, M.D., Ph.D.

